# A prostate-specific membrane antigen (PSMA)-targeted prodrug with a favorable in vivo toxicity profile

**DOI:** 10.1038/s41598-021-86551-1

**Published:** 2021-03-29

**Authors:** Srikanth Boinapally, Hye-Hyun Ahn, Bei Cheng, Mary Brummet, Hwanhee Nam, Kathleen L. Gabrielson, Sangeeta R. Banerjee, Il Minn, Martin G. Pomper

**Affiliations:** 1 Russell H. Morgan Department of Radiology and Radiological Science, Johns Hopkins Medical Institutions, Baltimore, MD USA; 2 Department of Molecular and Comparative Pathobiology, Johns Hopkins Medical Institutions, Baltimore, MD USA

**Keywords:** Cancer, Drug discovery, Chemistry

## Abstract

Prostate-specific membrane antigen (PSMA) is a promising target for the treatment of advanced prostate cancer (PC) and various solid tumors. Although PSMA-targeted radiopharmaceutical therapy (RPT) has enabled significant imaging and prostate-specific antigen (PSA) responses, accumulating clinical data are beginning to reveal certain limitations, including a subgroup of non-responders, relapse, radiation-induced toxicity, and the need for specialized facilities for its administration. To date non-radioactive attempts to leverage PSMA to treat PC with antibodies, nanomedicines or cell-based therapies have met with modest success. We developed a non-radioactive prodrug, SBPD-1, composed of a small-molecule PSMA-targeting moiety, a cancer-selective cleavable linker, and the microtubule inhibitor monomethyl auristatin E (MMAE). SBPD-1 demonstrated high binding affinity to PSMA (*K*_i_ = 8.84 nM) and selective cytotoxicity to PSMA-expressing PC cell lines (IC_50_ = 3.90 nM). SBPD-1 demonstrated a significant survival benefit in two murine models of human PC relative to controls. The highest dose tested did not induce toxicity in immunocompetent mice. The high specific targeting ability of SBPD-1 to PSMA-expressing tumors and its favorable toxicity profile warrant its further development.

## Introduction

Prostate-specific membrane antigen (PSMA) is over-expressed on the membrane of aggressive forms of prostate cancer (PC)^1,2^, other human cancers^3^, and endothelial cells of tumor neovasculature^1^. PSMA can also be engineered into T cells as a reporter for imaging or targeted killing^4,5^. Those attributes have made PSMA a highly leveraged marker for imaging and targeted therapy of PSMA-expressing tumors^6–9^ or cell-based therapies equipped with PSMA as a reporter^10^.

Radiopharmaceutical therapy (RPT) targeting advanced PC has been tested in clinical trials to good effect for patients who are refractory to currently approved therapies^11–13^. Despite those promising results, PSMA-targeted RPT still has limitations. RPT using beta-particle emitters, e.g., ^177^Lu, have enabled substantial imaging and prostate-specific antigen (PSA) responses with minimal side effects, but patients tend to relapse^14,15^. Clinical trials with alpha-particle emitters, e.g., ^225^Ac, have shown even better tumor responses, but also more severe toxicities including lethal renal failure in preclinical models, xerostomia, and alacrima^16–18^. Furthermore, administration of RPT requires specialized facilities for management of radioactivity. In part because of those shortcomings, PSMA-targeted therapies other than RPT are actively sought^19–21^.

The prodrug concept has been developed to avoid unwanted side effects of potent drugs with a narrow therapeutic window^22^. The prodrug itself is inactive and becomes the active pharmaceutical ingredient only through a specific interaction at the target site, such as through enzymatic cleavage of an ester or peptide bond. Although PSMA-targeted RPT has shown a degree of clinical success as noted above, an additional specificity-conferring mechanism beyond the over-expression of PSMA in malignant tissues may provide an even greater measure of safety, as PSMA is expressed in some normal tissues, notably kidney^23,24^. A similar concept has been tested in the form of an antibody–drug conjugate (ADC) using a humanized anti-PSMA monoclonal antibody conjugated to monomethyl auristatin E (MMAE) through a valine-citrulline linker^25,26^. MMAE is a very potent microtubule inhibitor used for an early ADC approved by the US FDA, Brentuximab vedotin^27^. Brentuximab vedotin used a valine-citrulline linker^28^ between the drug and the antibody, which is a dipeptide designed to be enzymatically cleaved by cathepsin B, a lysosomal protease over-expressed in malignant cells^29^. That PSMA ADC demonstrated a high therapeutic index in preclinical models of prostate tumors refractory to docetaxel^25^. A recent phase I trial, however, revealed that despite the prodrug approach the minimal effective dose (1.8 mg/kg) was too close to the maximum tolerated dose (2.5 mg/kg) and patients suffered from neutropenia, peripheral neuropathy, and an increase in liver transaminases^30^. The toxicity may have been due to an unfavorable pharmacokinetic profile of the administered antibody, such as prolonged circulation, resulting in accumulation of free drug, as has been observed in clinical studies with other ADCs^31^.

We synthesized (6*S*,9*S*,24*S*,28*S*)-1-amino-6-((4-((5S,8S,11S)-11-((*S*)-sec-butyl)-12-(2-((*S*)-2-((1*R*,2*R*)-3-(((1*S*,2*R*)-1-hydroxy-1-phenylpropan-2-yl)amino)-1-methoxy-2-methyl-3-oxopropyl)pyrrolidin-1-yl)-2-oxoethyl)-5,8-diisopropyl-4,10-dimethyl-3,6,9-trioxo-2,13-dioxa-4,7,10-triazatetradecyl)phenyl)carbamoyl)-9-isopropyl-1,8,11,18,26-pentaoxo-2,7,10,19,25,27-hexaazatriacontane-24,28,30-tricarboxylic acid (SBPD-1), a PSMA-targeted prodrug using a low-molecular-weight, urea-based PSMA targeting moiety conjugated to monomethyl auristatin E (MMAE) through a valine-citrulline linker. We evaluated its target specificity, serum stability, cytotoxicity against PSMA-expressing tumors, and in vivo toxicity.

## Results

### SBPD-1 binds with high affinity to PSMA and contains a cathepsin B cleavable linker.

**Figure 1:**
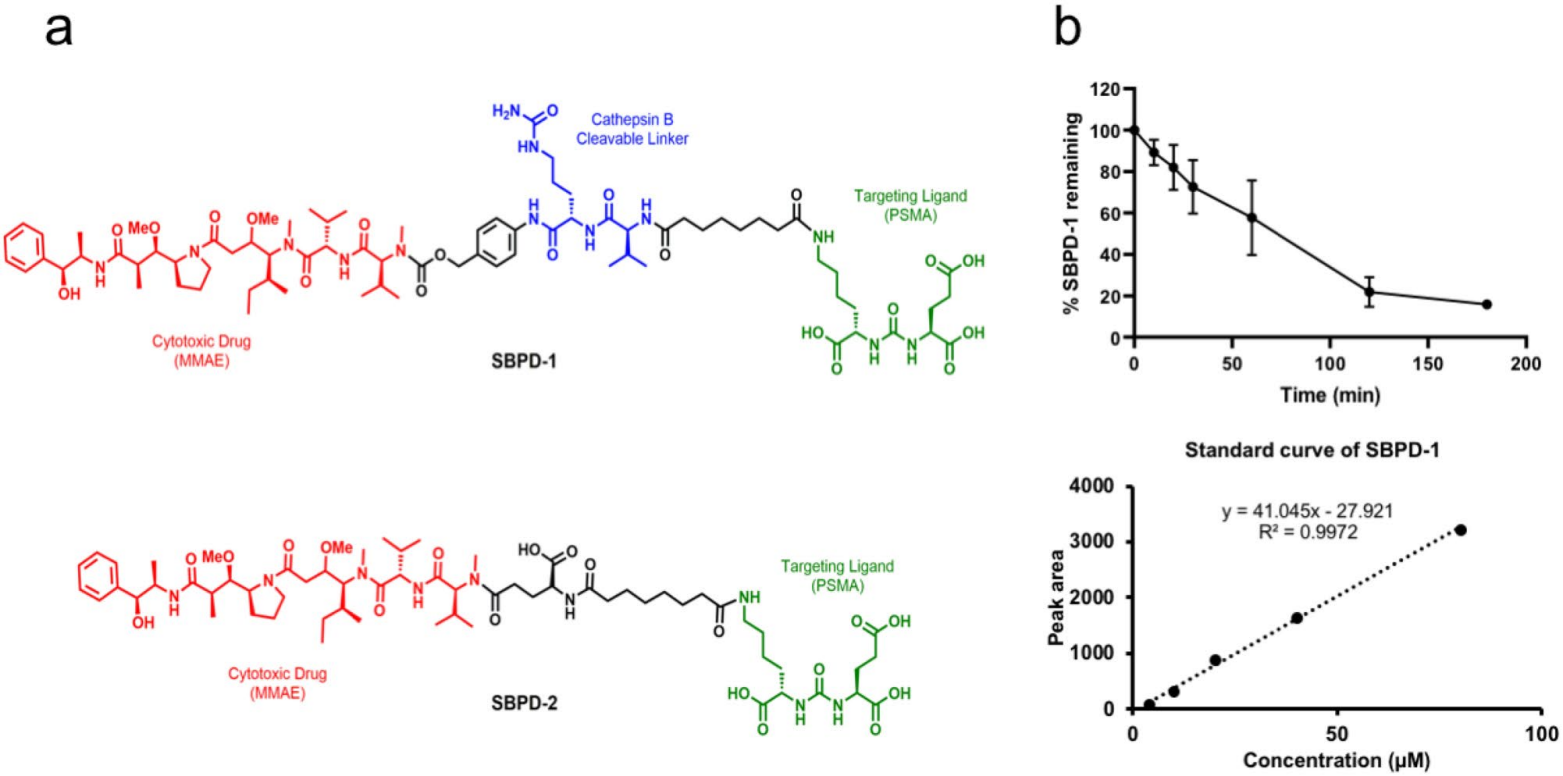
SBPD-1 is a PSMA-targeted prodrug that releases MMAE through the action of cathepsin B. (**a**) Structures of SBPD-1 and SBPD-2. PSMA-targeting moiety (green), linker (black), cathepsin B cleavable linker (Blue), and MMAE (red). (**b**) Release of MMAE upon treatment of SBPD-1 with recombinant cathepsin B represented by decrease of intact SBPD-1 (upper). Standard curve generated for the quantification of intact SBPD-1 (lower).

To achieve a specific, high-affinity interaction with PSMA we used the low-molecular-weight (LMW) scaffold Lys-Glu-Urea-DSS originally developed in our laboratory^32^. The synthetic tubulin inhibitor MMAE was conjugated to the Lys-Glu-Urea-DSS via a cathepsin B cleavable valine-citrulline linker (SBPD-1) or non-cleavable linker (SBPD-2), as a control to determine the utility of the linker (Fig. 1a).

Synthesis of SBPD-1 began with known amine **1**^33^, which on treatment with previously reported Lys-Glu-Urea-DSS^32^ in the presence of diisopropylethylamine afforded **2** in 85% yield (Supplementary Fig. S1). Compound **2** was further converted into activated carbonate **3** in 45% yield by treating with bis(4-nitrophenyl) carbonate and subsequent reaction with MMAE, followed by deprotection to realize target conjugate SBPD-1 in 20% combined yield (Supplementary Fig. S1).

Synthesis of SBPD-2 began with previously reported Lys-Glu-Urea-DSS^32^, which on treatment with L-glutamic acid α-tert-butyl ester in the presence of diisopropylethylamine in DMF, afforded **4** in 70% yield (Supplementary Fig. S2). Compound **4** was subsequently reacted with MMAE followed by deprotection to provide target conjugate SBPD-2 in 20% combined yield (Supplementary Fig. S2).

PSMA inhibitory capacity, a surrogate for affinity, was measured according to a previously described assay^34^. Both conjugates, SBPD-1 and SBPD-2, demonstrated high affinity to PSMA with *K*_i_ values of 8.84 nM (95% CI 5.00–15.63) and 3.0 nM (95% CI 1.94–4.67), respectively. We tested if SBPD-1 could release MMAE when incubated with recombinant cathepsin B in vitro and found that MMAE was efficiently released (80%) within 3 h of incubation (Fig. 1b).

### SBPD-1 selectively kills PSMA-expressing PC cells in vitro

**Figure 2:**
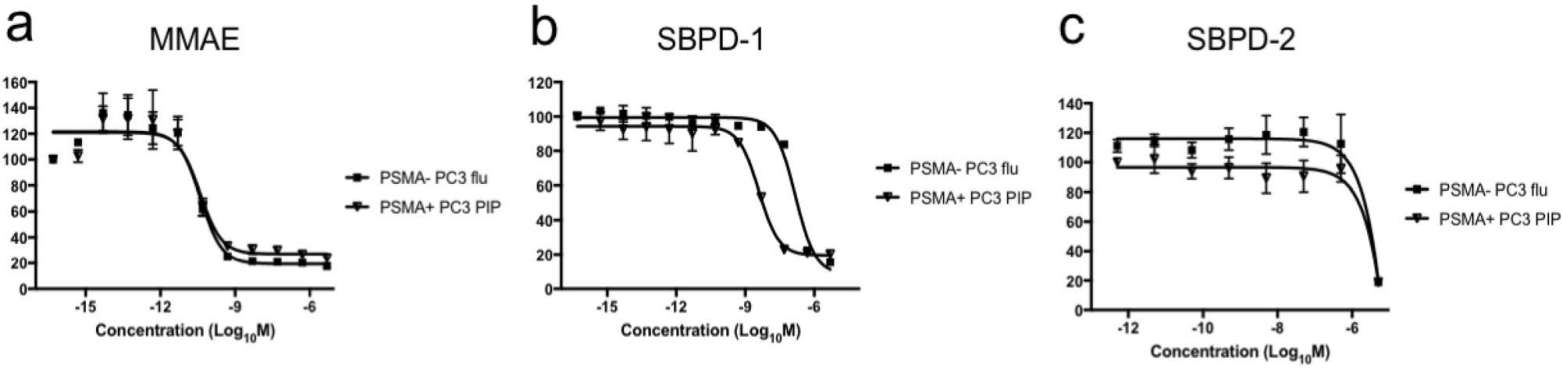
SBPD-1 selectively kills PSMA-expressing cancer cells. Sigmoidal curves of MMAE (**a**), SBPD-1 (**b**), and SBPD-2 (**c**) for cytotoxic activity against PSMA + PC3 PIP and PSMA − PC3 flu cell lines. Results were obtained at 48 h.

We evaluated the cytotoxicity of SBPD-1 and SBPD-2 in PSMA-expressing PC3 PIP and PSMA-negative PC3 flu cells in vitro^35,36^. SBPD-1 demonstrated IC_50_ values of 3.9 nM (95% CI 2.8–5.5 nM) and 151.1 nM (95% CI 104.1–219.3 nM) for PSMA + PC3 PIP and PSMA − PC3 flu cells, respectively, indicating selectivity for PSMA-expressing cells. The IC_50_ value of 151.1 nM for PSMA − PC3 flu cells suggests release of some MMAE to enable non-selective cell kill in vitro. SBPD-2 demonstrated IC_50_ values of 4.8 μM (95% CI 0.8–28.5 μM) and 5.8 μM (95% CI 0.7–47.2 μM) for PSMA + PC3 PIP and PSMA− PC3 flu cells, respectively, indicating a lack of potency regardless of PSMA expression and the need for cleavage of MMAE from the targeting moiety. MMAE alone proved exquisitely potent in both cell lines, demonstrating an IC_50_ value of 39.2 pM (95% CI 19.5–78.7 pM) and 40.0 pM (95% CI 21.2–75.4 pM) at 48 h for PSMA+ PC3 PIP and PSMA− PC3 flu cells, respectively (Fig. 2).

### SBPD-1 selectively kills PSMA-expressing PC xenografts in vivo

**Figure 3:**
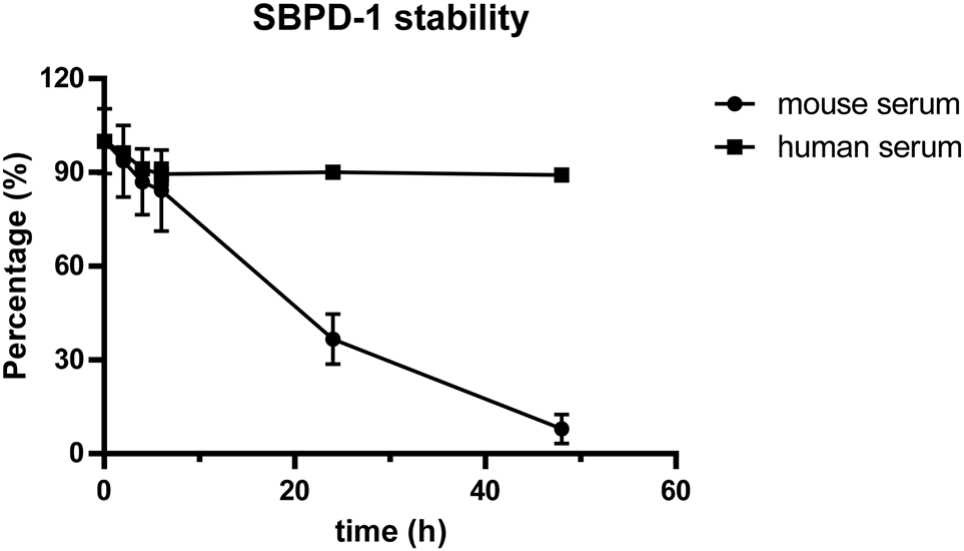
SBPD-1 is more stable in human than in murine serum. SBPD-1 was quantified by HPLC at various times after incubation with human or murine serum.

Prior to in vivo potency we evaluated the stability of SBPD-1 and SBPD-2 in human and murine serum. SBPD-1 remained intact in human serum out to 48 h of incubation (Fig. 3). While 90% of SBPD-2 remained intact for 48 h in murine serum (data not shown), SBPD-1 was metabolized more quickly (Fig. 3). While more than 80% of SBPD-1 was intact in serum at 8 h of incubation, less than half represented parent compound at 24 h, and the majority of the prodrug was fully degraded by 48 h of incubation. It has been reported that the valine-citrulline linker is stable in human and monkey serum but that it can be hydrolyzed in mouse plasma via extracellular carboxylesterase 1c^37,38^. Based on those stability results, we applied small, fractionated doses for the murine efficacy study to avoid systemic toxicity that could affect the overall survival of the test animals.

**Figure 4:**
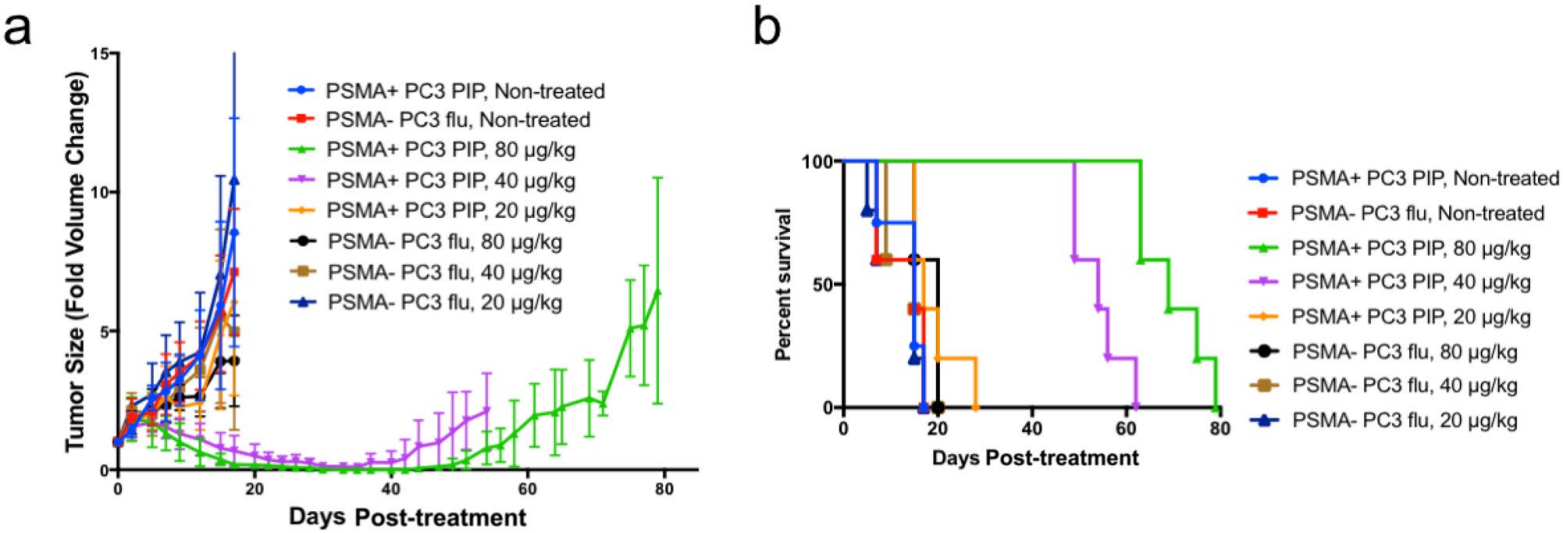
SBPD-1 selectively inhibited PSMA-expressing tumor growth in vivo. (**a**) Changes in size of PSMA + PC3 PIP and PSMA − PC3 flu subcutaneous tumors grown in NSG mice treated with varying doses of SBPD-1. (**b**) A fourfold increase in tumor volume was scored as death of an animal.

To evaluate efficacy in preclinical models of human PC, we initially employed xenograft tumor models derived from PSMA+ PC3 PIP and PSMA− PC3 flu cells in NOD/SCID/IL2Rγnull (NSG) mice. Three weeks after injection of the cells, the average tumor volume reached 62.4 (± 11.6) mm^3^, and mice were treated with 20, 40 and 80 μg/kg of SBPD-1 via daily intraperitoneal (IP) injection for 30 days, n = 5. We monitored tumor growth and overall animal welfare (Fig. 4a). Animals were scored ‘dead’ when the tumor reached 4-times its original volume (Fig. 4b). Tumors in non-treated, control mice for both tumor types, in PSMA+ PC3 PIP mice treated with 20 μg/kg and in PSMA− PC3 flu mice with all three doses, grew rapidly and all animals so treated were euthanized on day 20 post-initiation of treatment (Fig. 4b). The median survival time for non-treated groups of animals harboring either PSMA+ PC3 PIP or PSMA − PC3 flu tumors was 15 days. For animals harboring PSMA+ PC3 PIP tumors, the median survival time of the group treated with 20 μg/kg was 17 days. The median survival times for group harboring PSMA − PC3 flu tumors treated with 20, 40, and 80 μg/kg were 15, 15, and 20 days, respectively. Doses of 40 and 80 μg/kg delivered to animals harboring PSMA + PC3 PIP tumors cleared the tumors such that they were undetectable by the completion of treatment (Fig. 4a). Approximately 1 week was required to be able to re-measure previously undetectable tumors in the group treated at 40 μg/kg. Two weeks were required for re-appearance of tumors in animals treated with the 80 μg/kg dose. In animals harboring PSMA + PC3 PIP tumors, both the 40 and 80 μg/kg doses provided significant survival benefits as the median survival times were 54 days [*P* = 0.003, Log-rank (Mantel-Cox) test] and 69 days (*P* = 0.003), respectively (Fig. 4b). Urine protein level and specific gravity measured for all test animals on Days 9 and 20 were normal, indicating that no acute renal toxicity occurred at any dose tested (Supplementary Table S1).

### SBPD-1 is effective in an experimental metastatic model of PSMA-expressing PC

**Figure 5:**
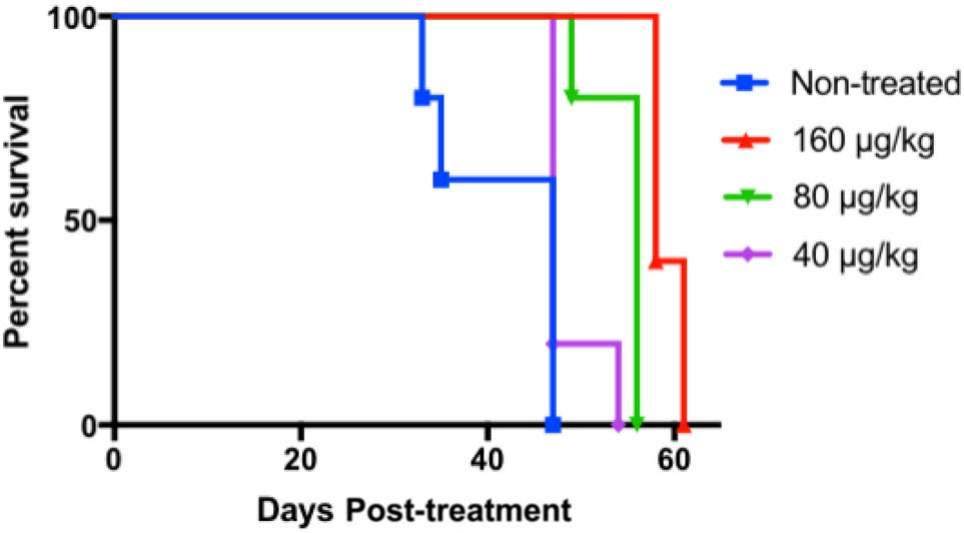
SBPD-1 provided a dose-dependent survival benefit in animals with metastatic PSMA + prostate cancer. Survival curves representing mice treated with the indicated doses of SBPD-1. Animals harbored metastatic tumors derived from PSMA + PC3/ML/PSMA cells administered intravenously.

To evaluate efficacy of SBPD-1 on established metastatic tumors, we used a PSMA-expressing experimental metastatic model of human PC^18^. The model has 100% penetrance, and consistently develops lesions in the liver (100%), kidney (100%), and bone (40%). PSMA + PC3/ML/PSMA cells were administered to NSG mice intravenously (IV) and tumors were allowed to establish for 4 weeks. PC3/ML/PSMA cells express firefly luciferase as an imaging reporter to allow us to monitor tumor development via weekly bioluminescence imaging (BLI). Mice were treated with 40, 80 and 160 μg/kg of SBPD-1 via daily IP injection for 30 days, n = 5. We increased the doses to compensate for the lower expression of PSMA on PC3/ML/PSMA cells compared with that of PSMA + PC3 PIP tumors (Supplementary Figure S3). The 40 μg/kg dose did not show survival benefit to non-treated control mice, with median survival times of 47 days for each group. Mice treated at the 80 and 160 μg/kg dose levels, however, exhibited significant survival benefits, with median survival of 56 days [*P* = 0.003, Log-rank (Mantel-Cox) test] and 58 days (*P* = 0.003), respectively (Fig. 5, Supplementary Figure S4).

### SBPD-1 is non-toxic to C57BL/6 mice

**Figure 6:**
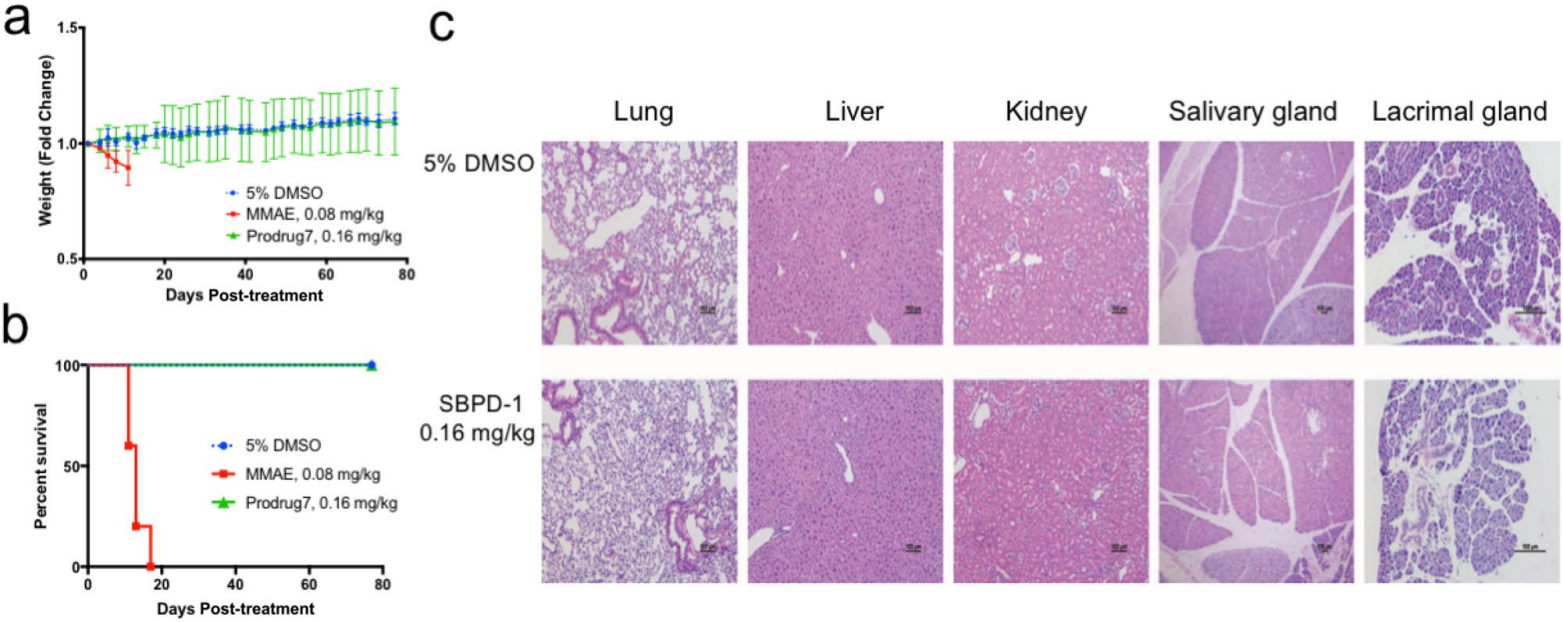
SBPD-1 is not toxic to healthy mice. (**a**) Changes in weight and (**b**) survival of CD-1 mice treated with the indicated drugs. (**c**) Representative histology of selected organs after the completion of the treatment with DMSO (vehicle) and SBPD-1 (scale bar 100 μm). No damage occurred within tissues tested.

We evaluated potential toxicity of SBPD-1 in immunocompetent animals. We administered MMAE (80 μg/kg), SBPD-1 (160 μg/kg), and 5% DMSO to healthy C57BL/6 mice (n = 5). We monitored animals for 80 days after initiation of administration. As previously reported^39^, MMAE demonstrated severe toxicity as all treated mice required euthanasia during treatment due to weight loss (Fig. 6b). Mice injected with vehicle or SBPD-1 did not show any signs of toxicity and steadily gained weight (Fig. 6a). We removed lung, liver, kidneys, salivary and lacrimal glands from all tested animals at Day 80 after initiation of SBPD-1 treatment. Histopathological examination revealed no tissue damage (Fig. 6c). We also obtained peripheral blood from mice injected with vehicle, SBPD-1, and healthy untreated animals, and prepared serum for chemistry studies (n = 5). Blood urea nitrogen (BUN), creatinine, glucose, alkaline phosphatase (ALP), total protein (T-Pro), and alanine aminotransferase (ALT) analyses showed that animals injected with either vehicle or SBPD-1 did not show differences in these values compared with those from untreated mice (Supplementary Table S2). Complete blood counts from the mice also showed no abnormalities except for lower white blood cell count for mice injected with SBPD-1, which may have resulted from the relative instability of the cathepsin B linker in murine serum and subsequent bone marrow toxicity of MMAE^37,40^.

## Discussion

Prostate-specific membrane antigen (PSMA) was first identified as a marker for PC through cloning of a monoclonal antibody raised against the patient-derived PC cell line, LNCaP^41^. Since PSMA was discovered to be the same as the *N*-acetyl-l-aspartyl-l-glutamate peptidase I (NAALADase I)^42^, PSMA has been pursed as a target for diagnostic imaging of advanced PC with various low-molecular-weight agents^35,43–46^. Anti-PSMA antibodies have also been tested as PSMA-targeting entities for both molecular imaging and therapy of PC^6–8,47,48^. Other therapeutic approaches such as PSMA targeted-nanoparticles loaded with an anti-cancer drug^49,50^ or photodynamic therapy^51–53^ have been tested in preclinical and clinical settings. PSMA-targeted RPT has provided a new alternative to managing patients with advanced PC refractory to other therapies^54,55^. Recent prospective trials of ^177^Lu-based therapies have demonstrated substantial imaging and PSA responses^56,57^. Fewer side effects than other systemic therapies, such as hormonal or chemotherapy, have repeatedly been shown^58^. Nevertheless, approximately 50% of patients were non-responders, and the majority of responders relapsed, requiring further cycles or other options^55^. Questions about long-term toxicity of this method remain, particularly for α-particle emitting versions of RPT^17,18,59,60^.

Although PSMA-targeted RPT is promising and fraught with fewer adverse events compared to the conventional cytotoxic therapies, radiation exposure to normal organs can result in xerostomia or other off-target effects^16–18,59,60^. A PSMA-targeted prodrug equipped with additional specificity to malignant cells may provide an enhanced therapeutic index. Several PSMA-targeted prodrugs were tested in both preclinical and clinical settings. Kularatne et al*.* tested various cytotoxic drugs as a form of prodrug by conjugating them to the PSMA-targeted agent, 2-[3-(1,3-dicarboxy propyl)ureido] pentanedioic acid^61^. Those prodrugs utilized a disulfide linker to enable drug release in the reducing environment of the cytoplasm. Some of the tested drugs exhibited cytotoxicity to PSMA-expressing LNCaP cells at single- or double-digit nanomolar concentration levels. However, in vivo safety and efficacy of those drugs have not been tested. Mipsagargin (G-202) is a prodrug consisting of an analog of thapsigargin conjugated to a PSMA-cleavable peptide^62^. Thapsigargin is a potent inhibitor of the sarcoplasmic/endoplasmic reticulum calcium adenosine triphosphatase (SERCA) pump essential for cell viability. Mipsagargin was used to target the PSMA-expressing tumor neovasculature of various solid cancers. Despite promising preclinical and phase I results^62^, phase II trials showed no clinical benefit for advanced hepatocellular carcinoma^63^. A PSMA-targeted antibody-MMAE conjugate (ADC) has been tested and showed favorable preclinical efficacy^25,26^. However, in a phase I trial with that conjugate, the therapeutic window proved narrow, necessitating modification of dose selection if the compound were to advance further^30^. The authors of that trial hypothesized that the toxicity may have been due to the systemic concentration of free MMAE released from the antibody^30^. The results from the corresponding phase II trial were recently published^64^. Toxicity was noted shortly after the initiation of the trial—particularly neutropenia and neuropathy—such that a dose reduction was necessary for it to continue. A partial radiologic response was obtained in only 2 of 119 participants, with none reporting a complete response^64^.

Please note that we used PSMA + PC3 PIP cells to generate subcutaneous tumors that may not precisely reflect the case as it may occur in patients. Although we did not measure the number of PSMA molecules per PSMA + PC3 PIP cell in the current study, we have previously shown there to be an order of magnitude higher PSMA expression in these cells than in LNCaP cells, which are patient-derived^18^. However, PSMA + PC3/ML/PSMA cells used for the metastatic model have comparable PSMA expression to that of LNCaP cells. Nevertheless, we used the PSMA + PC3 PIP/PSMA − PC3 flu cells to generate subcutaneous tumors in order to minimize the number of variables between cells used, as these lines are otherwise isogenic, and to see if any signal could be obtained in this proof-of-principal study. Future studies will explore tumor models that have a variety of levels of PSMA expression, including those that are more in line with what is seen in human specimens.

SBPD-1 was designed for safe delivery of the potent toxin MMAE to maximize its therapeutic index. There are three layers of specificity of this agent for malignant cells. First, there is high-affinity, specific PSMA targeting followed by internalization of drug-bound PSMA. Notably PSMA tends to localize to the centrosome upon internalization^65^ enabling it to deliver a drug that interrupts microtubule formation to the compartment in which it can be most effective. Second, MMAE is released only upon enzymatic cleavage by cathepsin B, which is upregulated in the lysosomes of cancer cells^29^. The same drug with non-cleavable linker (SBPD-2) showed about 7,100-fold less potency in PSMA + cancer cells (Fig. 2). Third, MMAE inhibits microtubule polymerization, an essential process for cell division of cancer cells. A further advantage of the small-molecule approach is that drug conjugates tend to have superior tumor penetration and more rapid clearance from non-target sites than do ADCs^66^.

Since prior reports^37,38^ as well as our results (Fig. 3a) have suggested that the valine-citrulline linker is unstable in murine serum, we modified the dosing plan to consist of several fractionated doses. Our in vivo safety results with an immunocompetent murine model showed no toxicity with the highest doses tested in the efficacy study (Fig. 6, Supplementary Table S2). It is likely that a clinical dosing plan could consist of less frequent administration as the valine-citrulline linker has been reported to be stable in human plasma^38^.

In summary, we have generated and tested in vivo a low-molecular-weight, PSMA-targeted prodrug that demonstrated tumor penetration and specificity sufficient to provide survival differences between PSMA + tumor-bearing animals and animals bearing isogenic tumors devoid of PSMA, including in a metastatic model. Furthermore, despite carrying the potent anti-tumor agent MMAE, the conjugate was non-toxic. We believe that lower toxicity was due to the controlled environment to which MMAE was delivered, by virtue of the presence of a cathepsin B cleavable linker in the molecule. Compounds of this class or those employing similar strategies may enable safe and effective targeting of PSMA-expressing lesions in patients.

## Methods

### General methods and materials for syntheses of prodrugs

Experiments were carried out in compliance with ARRIVE guidelines. Detailed methods for the syntheses of prodrugs are described in the Supplementary Information. Commercially available reagents and solvents for syntheses were analytical grade and used without further purification. Diisopropylethylamine (DIPEA), triflouroacetic acid (TFA), 4-(Dimethyl amino) pyridine (DMAP), pyridine (Py) and *N*-(3-dimethylaminopropyl)-*N*-ethylcarbodiimide (EDC) were purchased from Sigma-Aldrich (Allentown, PA, USA). l-Glutamic acid 5-tert-butyl ester, bis(4-nitrophenyl) carbonate and 1-hydroxybenzotriazole hydrate (HOBt) were purchased from Chem-Impex International (Wood Dale, IL, USA), disuccinimidyl suberate was purchased from TCI America (Pittsburgh, PA, USA) and monomethyl auristatin E (MMAE) was purchased from BroadPharm (San Diego, CA, USA). High performance liquid chromatographic (HPLC) purification of final compounds (SBPD-1 and SBPD-2) was performed using a C_18_ Luna 10 mm × 250 mm column (Phenomenex, Torrance, CA, USA) on an Agilent 1260 infinity LC system (Santa Clara, CA, USA) and eluted with water (0.1% TFA) (A) and CH_3_CN (0.1% TFA) (B). ^1^H NMR spectra were recorded on a Bruker Ultrashield 500 MHz spectrometer. Chemical shifts (δ) are reported in parts per million (ppm) downfield by reference to proton resonances resulting from incomplete deuteration of the NMR solvent and the coupling constants (J) was reported in Hertz (Hz). High resolution mass spectra were obtained by the University of Notre Dame Mass Spectrometry and Proteomics Facility, Notre Dame, IN using ESI by direct infusion on a Bruker micrOTOF-II.

### Cathepsin B cleavage

Release of MMAE from prodrugs by a recombinant cathepsin B was analyzed using a modified method from previously published work^33^. Prodrug stock solutions (80 µL, 10 mM) were added to the 1.92 mL cathepsin B (MilliporeSigma, Cat# C8571, Burlington, MA, USA) containing buffer (25 mM acetate, 1 mM EDTA, pH 5, pre-warmed at 37 °C) at the final concentration of 30 nM (cathepsin B)and 40 µM (prodrug). Aliquots (200 µL) were periodically removed and enzymatic activity was stopped by the addition of thioprotease inhibitor E-64 (30 nM in the final solution, MilliporeSigma, Cat# E3132). The samples were centrifuged and the supernatants were analyzed by HPLC (Waters 600 E coupled with Varian prostar detector, Milford, MA, USA). Samples were prepared at 0, 10, 20, 30, 60, and 120 min. Experiments were performed in triplicate.

### PSMA affinity and in vitro cytotoxicity

PSMA affinities of SBPD-1and SBPD-2 were measured using the modified Amplex Red glutamic acid/glutamate oxidase assay as previously described^34^. PSMA-expressing PC3-PIP, PSMA-negative PC3-flu, PSMA-positive PC3/ML/PSMA and PSMA-negative PC3/ML were maintained as previously described^18^. One thousand cells (PC3-PIP or PC3-flu) were seeded in 96 well plates 24 h prior to drug treatment. Drug was added to each well in serial dilution and incubated for 24, 48 or 72 h. Cell viability was measured using TACS XTT Cell Proliferation Assay (Trevigen, Cat# 4891-25-K, Gaithersburg, MD) at each time point according to the manufacturer’s protocol. IC_50_ values were calculated using GraphPad Prism 7 software.

### Serum stability

Serum stability of prodrugs was analyzed using a modified method from previously published work^67^. Prodrug stock solution (80 µL, 1 mM) was mixed with human serum (320 µL) purchased from Millipore Sigma (Cat# H4522, Saint Louis, MO, USA). Five 50 µL fractions corresponding to five-time points (2, 4, 6, 24, and 48 h) were removed in separate vials from the above mixture and incubated at 37 °C. Aliquots of 25 µL were removed at 2, 4, 6, 24, and 48 h from the respective vial and diluted with cold ice CH_3_OH (125 µL) to precipitate proteins. The samples were centrifuged, and the supernatants were analyzed by HPLC [λ 220 nm, 250 mm × 4.6 mm Phenomenex Luna C18 column, solvent gradient: 61% H2O (0.1% TFA) and 39% ACN (0.1% TFA) isocratic for 30 min at a flow rate of 1 mL/min. SBPD-1 eluted at 12.1 min]. Murine prodrug stock solution (32 µL, 10 mM) was incubated with 100% mouse serum (final concentration of the serum was 80% after the mixing with prodrug solution) at 37 °C. Proteins were precipitated as above. Aliquots of 25 µL were evaluated at the same time points as above. The samples were centrifuged, and the supernatants were analyzed by HPLC as above. Stability was calculated based on the peak area of the prodrug at each time point. Experiments were performed in triplicate.

### Preclinical evaluation of SBPD-1

Animal studies were performed under the guidance of a protocol approved by the Johns Hopkins Animal Care and Use Committee and performed in compliance with the Animal Welfare Act regulations and Public Health Service (PHS) Policy. Johns Hopkins University has an approved PHS assurance. NSG (NOD/SCID/IL2Rγnull) mice were purchased from the Johns Hopkins University Sydney Kimmel Comprehensive Cancer Center Animal Resources Core. C57BL/6 mice were purchased from Jackson Laboratory (Bar Harbor, ME, USA). NSG mice were injected with 1.5 million PC3/PIP or 1 million PC3/flu cells at the lower left flank. Two weeks after the injection of cells, mice were treated with 20, 40, 80 μg/kg of SBPD-1 formulated in 100 μL of sterile saline via daily intraperitoneal (IP) injection for 30 days. Tumor volumes were measure twice per week. Urinalysis was performed using URS-10 Urine Reagent Strips (LW Scientific Inc. Lawrenceville, GA).

For the metastatic model, NSG mice were injected with 0.75 million PC3/ML/PSMA cells via the tail vein. Four weeks after the injection mice were treated with 40, 80, 160 μg/kg of SBPD-1 formulated in 100 μL of sterile water via daily intraperitoneal injection for 30 days. BLI was performed weekly using the IVIS Spectrum in vivo imaging system (Perkin Elmer, Waltham, MA).

### In vivo toxicity

Male C57BL/6 mice were purchased from Jackson Laboratory. Ten-week-old mice were injected with the indicated doses of MMAE (formulated in 5% DMSO), SBPD-1 (formulated in saline) or 5% DMSO intraperitoneally (daily for 30 days, n = 5). Animals were monitored daily for weight changes and other abnormalities for 80 days. Animals were euthanized in a CO_2_ chamber at day 80, and blood, lung, liver, kidney, salivary gland, and lacrimal gland were collected for complete blood counts, blood chemistry, and histopathological analyses. Complete blood counts including white blood cells (WBC), red blood cells (RBC), hemoglobin (HGB), hematocrit (HCT), mean corpuscular volume (MCV), mean corpuscular hemoglobin (MCH), mean corpuscular hemoglobin concentration (MCHC), and platelet (PLT) were measured using scil Vet ABC Hematology Analyzer (scil animal care company, Gurnee, IL). Blood chemistry parameters including blood urea nitrogen (BUN), glucose (GLU), Alkaline Phosphatase (ALP), total protein (T-Pro), Alanine aminotransferase (ALT) and Creatinine (Cre) were measured with Spotchem EZ chemistry analyzer (Arkray USA, Edina, MN). Hematoxylin and eosin slides were generated for five organs and examined by certified veterinary pathologist.

## Supplementary Information

Supplementary Information

## Data Availability

All data used in this submission are included in the body of manuscript or in the Supplementary Information. Additional data related to the paper are available upon request.
