# Supplementary material for: A prostate-specific membrane antigen (PSMA)-targeted prodrug with a favorable in vivo toxicity profile
Source: Sci Rep. 2021 Mar 29;11:7114. doi: 10.1038/s41598-021-86551-1 (PMC8007718; doi:10.1038/s41598-021-86551-1)
Supplement: Supplementary file 1 — Supplementary Information [file 41598_2021_86551_MOESM1_ESM.docx]

**Supplementary Information**

**A Prostate-specific membrane antigen (PSMA)-targeted prodrug with favorable *in vivo* toxicity profiles**

Srikanth Boinapally^1,*^, Hye-Hyun Ahn^1,*^, Bei Cheng^1^, Mary Brummet^1^, Hwanhee Nam^1^, Kathleen L. Gabrielson^2^, Sangeeta R. Banerjee^1^, Il Minn^1^, Martin G. Pomper^1†^

**﻿**^1^Russell H. Morgan Department of Radiology and Radiological Science, Johns Hopkins Medical Institutions, Baltimore, Maryland, ^2^Department of Molecular and Comparative Pathobiology, Johns Hopkins Medical Institutions, Baltimore, Maryland

*These authors contributed equally to this work.

^†^Correspondence: Martin G. Pomper, M.D., Ph.D.

Johns Hopkins Medical School

601 N. Caroline Street

JHOC 3223

Baltimore, MD 21287

Email: [mpomper@jhmi.edu](mailto:mpomper@jhmi.edu)

**
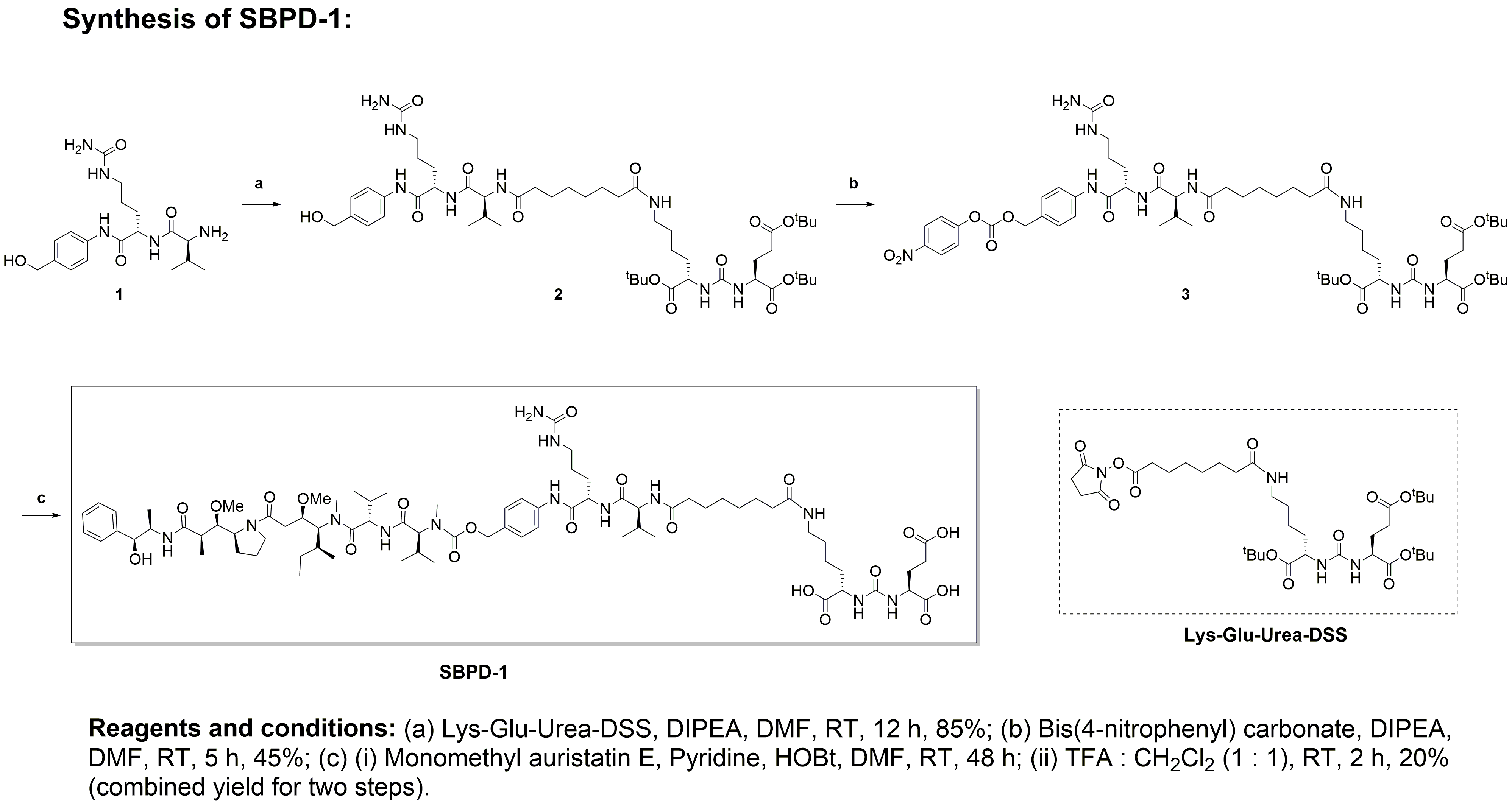
Experimental procedures and Spectral Characterization:**

**Supplementary Figure S1**

**Tri-*tert*-butyl (6*S*,9*S*,24*S*,28*S*)-1-amino-6-((4-(hydroxymethyl)phenyl)carbamoyl)-9-isopropyl-1,8,11,18,26-pentaoxo-2,7,10,19,25,27-hexaazatriacontane-24,28,30-tricarboxylate (2):** To a stirred solution of amine **1** (38 mg, 0.1 mmol, 1.0 eq.) and di-tert-butyl (((*S*)-1-(*tert*-butoxy)-6-(8-((2,5-dioxopyrrolidin-1-yl)oxy)-8-oxooctanamido)-1-oxohexan-2-yl)carbamoyl)-L-glutamate (74 mg, 0.1 mmol, 1.0 eq.) in dimethylformamide (1.0 mL) was added diisopropylethylamine (52 μL, 0.3 mmol, 3.0 eq.) at room temperature. The resulted mixture was stirred for 12 h at room temperature and concentrated in *vacuo*. The thick residue was triturated with diethyl ether (20 mL) for 30 minutes, and the solid was collected by filtration and washed with diethyl ether to obtain compound **2** as a colorless solid (85.5 mg, 85%). H^1^-NMR (500 MHz, DMSO-d_6_): δ 9.89 (s, 1H), 8.05 (d, J = 7.5 Hz, 1H), 7.81 (d, J = 8.5 Hz, 1H), 7.77-7.69 (m, 1H), 7.53 (d, J = 8.0 Hz, 2H), 7.22 (d, J = 8.0 Hz, 2H), 6.35-6.21 (m, 2H), 6.05-5.96 (m, 1H), 5.41 (s, 2H), 5.13 (t, J = 5.0 Hz, 1H), 4.42 (d, J = 4.5 Hz, 2H), 4.38-4.31 (m, 1H), 4.17 (t, J = 7.5 Hz, 1H), 4.06-3.97 (m, 1H), 3.98-3.90 (m, 1H), 3.15-3.07 (m, 2H), 3.06-2.95 (m, 2H), 2.95-2.91 (m, 1H), 2.31-2.08 (m, 4H), 2.06-1.79 (m, 4H), 1.74-1.52 (m, 4H), 1.53-1.30 (m, 14H), 1.38 (s, 27H), 0.83 (dd, J = 6.5, 7.5 Hz, 6H); HRMS (ESI) m/z: [M + H]+ calcd for C_50_H_85_N_8_O_13_, 1005.6226; found, 1005.6230.

**Tri-*tert*-butyl (6*S*,9*S*,24*S*,28*S*)-1-amino-9-isopropyl-6-((4-((((4-nitrophenoxy) carbonyl)oxy) methyl)phenyl)carbamoyl)-1,8,11,18,26-pentaoxo-2,7,10,19,25,27-hexaazatriacontane-24,28,30-tricarboxylate (3):** To a stirred solution of compound **2** (43 mg, 0.042 mmol, 1.0 eq.) and bis(4-nitrophenyl) carbonate (26 mg, 0.085 mmol, 2.0 eq.) in dimethylformamide (1.5 mL) was added diisopropylethylamine (20 μL, 0.085 mmol, 3.0 eq.) at room temperature under nitrogen atmosphere. The resulted mixture was stirred for 5 h at room temperature and concentrated in *vacuo*. The residue was triturated with diethyl ether (20 mL) for 1 h, and the solid was collected by filtration and washed with diethyl ether to obtain compound **3** as a brown color solid (22.5 mg, 45%). H^1^-NMR (500 MHz, DMSO-d_6_): δ 10.05 (s, 1H), 8.30 (d, J = 9.0 Hz, 2H), 8.15-8.06 (m, 1H), 7.80 (d, J = 8.0 Hz, 1H), 7.73 (t, J = 5.0 Hz, 1H), 7.64 (d, J = 8.5 Hz, 2H), 7.55 (d, J = 9.5 Hz, 2H), 7.40 (d, J = 8.5 Hz, 2H), 6.27 (dd, J = 8.5, 16.5 Hz, 2H), 6.05-5.94 (m, 1H), 5.42 (s, 2H), 5.23 (s, 2H), 4.40-4.32 (m, 1H), 4.22-4.13 (m, 1H), 4.06-3.98 (m, 1H), 3.97-3.90 (m, 1H), 3.09-2.88 (m, 5H), 2.28-2.06 (m, 4H), 2.05-1.80 (m, 4H), 1.75-1.54 (m, 4H), 1.54-1.41 (m, 8H), 1.37 (s, 27H), 1.29-1.14 (m, 6H), 0.90-0.77 (m, 6H); HRMS (ESI) m/z: [M + H]+ calcd for C_57_H_88_N_9_O_17_, 1170.6264; found, 1170.6292.

**(6*S*,9*S*,24*S*,28*S*)-1-Amino-6-((4-((5*S*,8*S*,11*S*,12*R*)-11-((*S*)-sec-butyl)-12-(2-((*S*)-2-((1*R*,2*R*)-3-(((1*S*,2*R*)-1-hydroxy-1-phenylpropan-2-yl)amino)-1-methoxy-2-methyl-3-oxopropyl)pyrrolidin-1-yl)-2-oxoethyl)-5,8-diisopropyl-4,10-dimethyl-3,6,9-trioxo-2,13-dioxa-4,7,10-triazatetradecyl)phenyl)carbamoyl)-9-isopropyl-1,8,11,18,26-pentaoxo-2,7,10,19,25,27-hexaazatriacontane-24,28,30-tricarboxylic acid (SBPD-1):** To a stirred solution of compound **3** (19.9 mg, 0.0169 mmol) and MMAE (9.0 mg, 0.0125 mmol) in dimethylformamide (800 μL) was added 1-hydroxybenzotriazole hydrate (0.33 mg, 0.0025 mmol) and pyridine (200 μL) at room temperature. The resulted mixture was stirred for 48 h at room temperature and concentrated in *vacuo*. The obtained residue was dissolved in 50% TFA in dichloromethane (2 mL) and resulted mixture was stirred at room temperature for 2 h followed by concentrated in *vacuo.* The crude was purified by preparative RP-HPLC chromatography using 0.1% TFA in H_2_O and 0.1% TFA in acetonitrile as eluents followed by lyophilization afforded target compound (SBPD-1) as a colorless solid (5.3 mg, 20%). [RP-HPLC purification was achieved using Agilent System, λ 254 nm, 250 mm x 10 mm Phenomenex Luna C_18_ column, solvent gradient: 80% H_2_O (0.1% TFA) and 20% ACN (0.1% TFA), reaching 60% of ACN in 20 min at a flow rate of 10 mL/min, product eluted at 14.4 min]. H^1^-NMR (500 MHz, DMSO-d_6_): δ 9.98 (s, 1H), 8.33-7.99 (m, 1H), 8.09 (d, J = 7.0 Hz, 1H), 7.93-7.52 (m, 5H), 7.39-7.11 (m, 7H), 6.30 (dd, J = 8.5, 16.5 Hz, 2H), 5.99 (s, 1H), 5.55-5.29 (m, 2H), 5.13-4.91 (m, 2H), 4.67-2.78 (m, 32H, merged in moisture peak), 2.43-1.87 (m, 12H), 1.85-1.15 (m, 25H), 1.09-0.68 (m, 31H); HRMS (ESI) m/z: [M + Na]+ calcd for
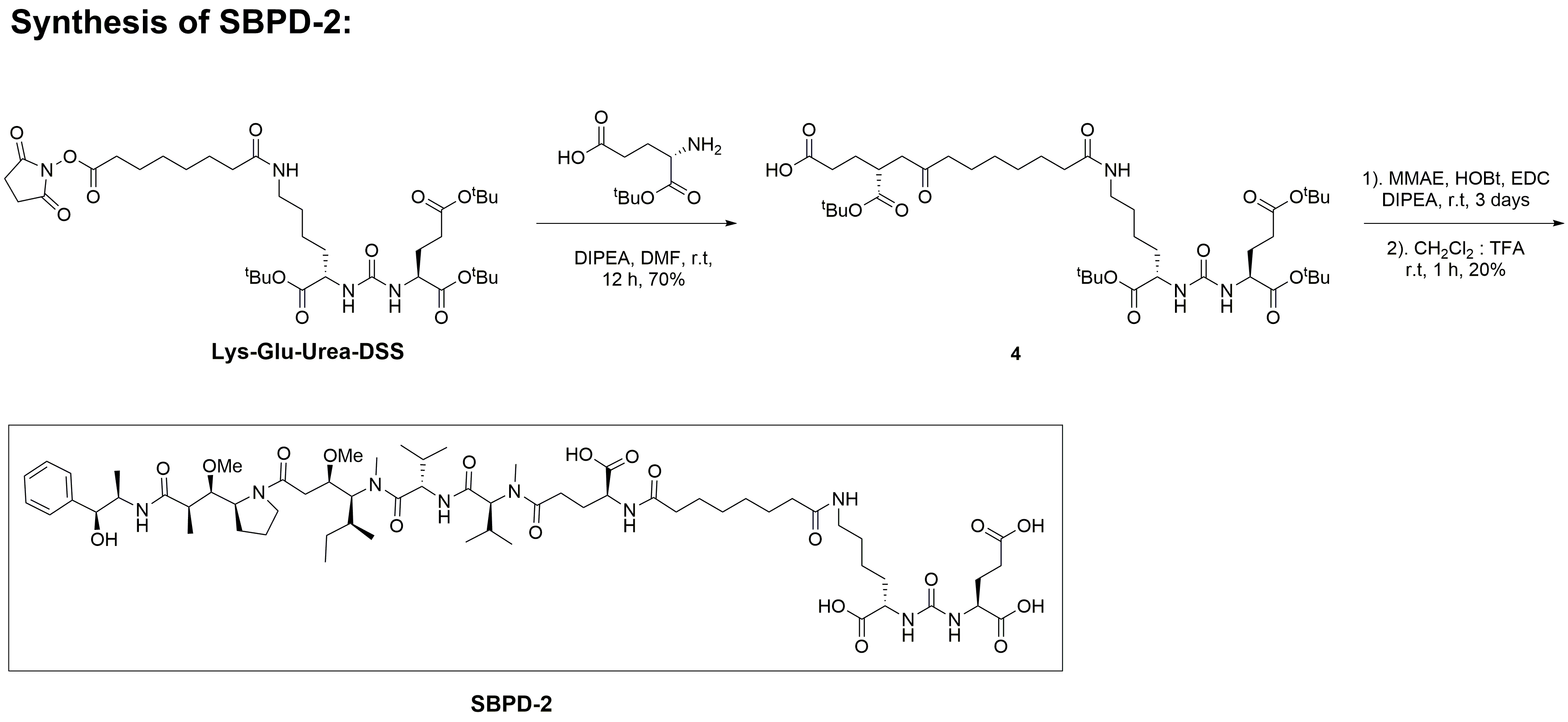
C_78_H_125_N_13_NaO_21_, 1602.9009; found, 1602.9005.

**Supplementary Figure S2**

**(7*S*,11*S*,26*R*)-7,26-Bis(*tert*-butoxycarbonyl)-11-carboxy-2,2-dimethyl-4,9,17,24-tetraoxo-3-oxa-8,10,16,25-tetraazanonacosan-29-oic acid (4):** To a stirred solution of (*S*)-4-amino-5-(tert-butoxy)-5-oxopentanoic acid (41 mg, 0.202 mmol, 1.5 eq) and di-*tert*-butyl (((*S*)-1-(*tert*-butoxy)-6-(8-((2,5-dioxopyrrolidin-1-yl)oxy)-8-oxooctanamido)-1-oxohexan-2-yl)carbamoyl)-L-glutamate (100 mg, 0.135 mmol, 1.0 eq) in dimethyl formamide (1.0 mL) was added diisopropylethylamine (95 μL, 0.539 mmol, 4.0 eq) at room temperature. The resulted mixture was stirred for 12 h at room temperature and concentrated in *vacuo*. The residue was purified by using ACN/H_2_O on C_18_ Sep-Pak column to provide compound **4** (78 mg, 70%) as a colorless solid. H^1^-NMR (500 MHz, CDCl_3_): *δ* 6.97-6.88 (m, 1H), 6.78 (d, *J* = 8.0 Hz, 1H), 6.02-5.70 (m, 2H), 4.53-4.44 (m, 1H), 4.34-4.17 (m, 2H), 3.33-3.12 (m, 2H), 2.50-2.14 (m, 8H), 2.09-1.93 (m, 2H), 1.87-1.70 (m, 2H), 1.70-1.25 (m, 14H), 1.45 (s, 9H), 1.44 (s, 9H), 1.43 (s, 9H), 1.41 (s, 9H).

**(4*S*,7*S*,10*S*,15*S*,30*S*,34*S*)-4-((*S*)-Sec-butyl)-3-(2-((*S*)-2-((1R,2R)-3-(((1*S*,2*R*)-1-hydroxy-1-phenylpropan-2-yl)amino)-1-methoxy-2-methyl-3-oxopropyl)pyrrolidin-1-yl)-2-oxoethyl)-7,10-diisopropyl-5,11-dimethyl-6,9,12,17,24,32-hexaoxo-2-oxa-5,8,11,16,25,31,33-heptaazahexatriacontane-15,30,34,36-tetracarboxylic acid (SBPD-2):** To a stirred solution of compound **4** (9.5 mg, 0.0114 mmol) and MMAE (8.2 mg, 0.0114 mmol) in dichloromethane (1.0 mL) was added HOBt (1.7 mg, 0.0125 mmol), EDC (2.41 mg, 0.0125 mmol) and DIPEA (6 μL, 0.0343 mmol) at room temperature. The resulted mixture was stirred for 3 days at room temperature and concentrated in *vacuo*. The obtained residue was dissolved in 50% TFA in dichloromethane (2 mL) and resulted mixture was stirred at room temperature for 1 h followed by concentrated in *vacuo.* The crude was purified by preparative RP-HPLC chromatography using 0.1% TFA in H_2_O and 0.1% TFA in acetonitrile as eluents followed by lyophilization afforded target compound (SBPD-2) as a colorless solid (3 mg, 20%). [RP-HPLC purification was achieved using Agilent System, λ 220 nm, 250 mm x 10 mm Phenomenex Luna C_18_ column, solvent gradient: 90% H_2_O (0.1% TFA) and 10% ACN (0.1% TFA), reaching 60% of ACN in 20 min at a flow rate of 10 mL/min, product eluted at 14.2 min]. H^1^-NMR (500 MHz, DMSO-*d_6_*): *δ* 13.20-11.72 (bs, 3H), 8.59-8.49 (m, 0.5H), 8.10-7.96 (m, 1H), 7.93-7.77 (m, 1H), 7.71 (t, *J* = 5.0 Hz, 1H), 7.62 (d, *J* = 8.0 Hz, 0.5H), 7.35-7.22 (m, 4H), 7.20-7.13 (m, 1H), 6.36-6.24 (m, 2H), 4.79-4.60 (m, 1H), 4.53-4.38 (m, 2H), 4.27-3.94 (m, 7H), 3.28-3.07 (m, 10H), 3.02-2.79 (m, 6H), 2.43-2.21 (m, 7H), 2.13-1.99 (m, 7H), 1.92-1.60 (m, 9H), 1.53-1.16 (17H), 1.07-0.97 (m, 6H), 0.87-0.68 (m, 18H); HRMS (ESI) m/z: [M + H]+ calcd for C_65_H_108_N_8_O_19_, 1304.7735; found, 1304.7725.

HPLC Report of SBPD-1:

[RP-HPLC report using Agilent System, l 254 nm, 250 mm x 10 mm Phenomenex Luna C18 column, solvent gradient: 80% H_2_O (0.1% TFA) and 20% ACN (0.1% TFA), reaching 60% of ACN in 20 min at a flow rate of 10 mL/min, product eluted at 14.4 min.]

HRMS Spectra of SBPD-1:


H^1^ NMR Spectra of SBPD-1 in DMSO-d_6_:

HPLC Report of SBPD-2:

[RP-HPLC report using Agilent System, λ 220 nm, 250 mm x 10 mm Phenomenex Luna C18 column, solvent gradient: 90% H_2_O (0.1% TFA) and 10% ACN (0.1% TFA), reaching 60% of ACN in 20 min at a flow rate of 10 mL/min, product eluted at 14.2 min].

HRMS Report of SBPD-2:

^1^HNMR Spectra of SBPD-2 in DMSO-d_6_

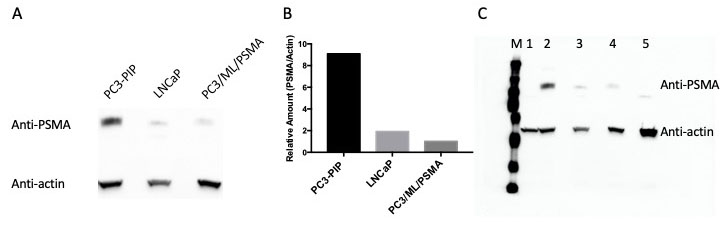


**Supplementary Figure S3**. PSMA expression levels in different cell lines varies.

(A) Western Blot and (B) quantified band intensities (PSMA band intensities were referenced by beta actin band intensities for relative amounts comparison). Western blot analysis of PSMA in different cell lines. Cells were harvested, sonicated in PBS, and centrifuge 13200 rpm for 15 min. Collected supernatant were resolved with SDS-PAGE and transferred to nitrocellulose membrane. The membrane was blocked with 5 % BSA in TBS-T and incubated overnight at 4 °C with primary antibodies (PSMA: Cell signaling, Cat# 12815; Beta actin: Santa Cruz Biotechnology cat# sc47778). After incubating with secondary antibody for 1 h, membrane was visualized by chemiluminescence method using Clarity Western ECL Substrate (Bio-Rad). Images were obtained using Gel Doc XR+ system (BIO-RAD). The intensity of each band was quantified using Volume Tools in Image Lab 6.0.1 (Bio-Rad). (C) Original (non-cropped) western blot of (A) is presented to meet the policy for digital image integrity and standards of the publisher. M: Mw marker, 1: PSMA-negative PC3-flu, 2: PSMA-positive PC3-PIP, 3: PSMA-positive LNCaP, 4. PSMA-positive PC3/ML/PSMA. 5: truncated-PSMA positive cell line. One PAGE gel was prepared and blotted on to one Immuno-Blot PVDF membrane (BIO-RAD). The PVDF membrane was cut horizontally in the middle of the membrane and the upper and lower membranes were stained for PSMA and actin, respectively. After completion of the Ab stainings, membranes were combined together and was visualized by chemiluminescence method as described above simultaneously.


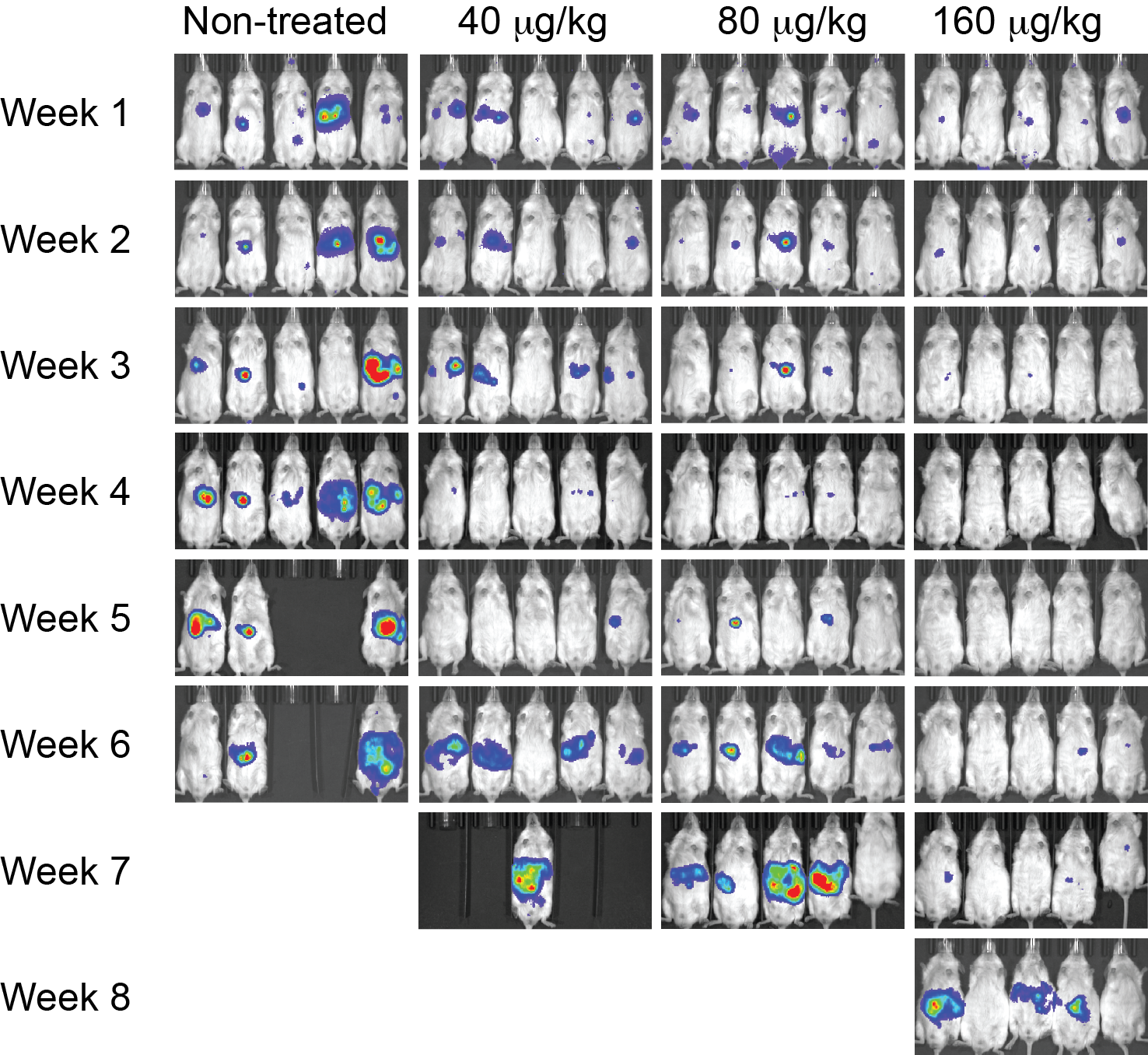


**Supplementary Figure S4**. Bioluminescence images of an experimental metastatic model of PSMA-expressing PC treated with SBPD-1. Treated doses are listed on top of the images.

Table S1. Urinalyses results from subcutaneous xenograft model.

| **Day 9** | | | | | |
| --- | --- | --- | --- | --- | --- |
|  |  | **PC3/PIP** | | **PC3/flu** | |
| **Treatment** | **Mouse #** | **protein** | **specific gravity** | **protein** | **specific gravity** |
| Untreated | Mouse 1 | 30 | 1.025 | trace | 1.025 |
|  | Mouse 2 | 30 | 1.025 | 30 | 1.025 |
|  | Mouse 3 | ND | ND | 30 | 1.025 |
|  | Mouse 4 | 30 | 1.025 | 30 | 1.025 |
|  | Mouse 5 | 30 | 1.025 | 30 | 1.03 |
| SBPD-1 0.08 mg/kg | Mouse 1 | trace | 1.025 | 300 | 1.025 |
|  | Mouse 2 | 30 | 1.025 | 30 | 1.025 |
|  | Mouse 3 | 30 | 1.025 | 300 | 1.025 |
|  | Mouse 4 | 30 | 1.025 | 100 | 1.025 |
|  | Mouse 5 | 30 | 1.025 | 30 | 1.025 |
| SBPD-1 0.04 mg/kg | Mouse 1 | 30 | 1.025 | 30 | 1.025 |
|  | Mouse 2 | trace | 1.025 | 30 | 1.025 |
|  | Mouse 3 | trace | 1.025 | 30 | 1.025 |
|  | Mouse 4 | 30 | 1.025 | 30 | 1.025 |
|  | Mouse 5 | 30 | 1.025 | 30 | 1.025 |
| SBPD-1 0.02 mg/kg | Mouse 1 | 30 | 1.025 | 30 | 1.025 |
|  | Mouse 2 | 30 | 1.02 | 30 | 1.025 |
|  | Mouse 3 | 30 | 1.025 | 30 | 1.025 |
|  | Mouse 4 | 30 | 1.025 | 30 | 1.02 |
|  | Mouse 5 | 30 | 1.02 | 30 | 1.025 |
| **Day 20** | | | | | |
|  |  | **PC3/PIP** | | **PC3/flu** | |
| **Treatment** | **Mouse #** | **protein** | **specific gravity** | **protein** | **specific gravity** |
| Untreated | Mouse 1 | 30 | 1.025 | trace | 1.025 |
|  | Mouse 2 | 30 | 1.025 | trace | 1.03 |
|  | Mouse 3 | ND | ND | 30 | 1.03 |
|  | Mouse 4 | 30 | 1.025 | 30 | 1.025 |
|  | Mouse 5 | 30 | 1.025 | 30 | 1.03 |
| SBPD-1 0.08 mg/kg | Mouse 1 | 30 | 1.025 | 300 | 1.025 |
|  | Mouse 2 | 30 | 1.025 | 30 | 1.025 |
|  | Mouse 3 | 100 | 1.025 | 300 | 1.025 |
|  | Mouse 4 | 30 | 1.025 | 30 | 1.025 |
|  | Mouse 5 | 100 | 1.025 | 30 | 1.025 |
| SBPD-1 0.04 mg/kg | Mouse 1 | 30 | 1.025 | 30 | 1.025 |
|  | Mouse 2 | 30 | 1.025 | 30 | 1.03 |
|  | Mouse 3 | 30 | 1.025 | ND | ND |
|  | Mouse 4 | 30 | 1.025 | 30 | 1.03 |
|  | Mouse 5 | 30 | 1.025 | 30 | 1.025 |
| SBPD-1 0.02 mg/kg | Mouse 1 | 30 | 1.025 | 30 | 1.025 |
|  | Mouse 2 | 30 | 1.025 | 30 | 1.01 |
|  | Mouse 3 | 30 | 1.025 | 30 | 1.025 |
|  | Mouse 4 | 30 | 1.025 | 30 | 1.03 |
|  | Mouse 5 | 30 | 1.025 | 30 | 1.025 |

ND: Not determined.

Table S2. Blood chemistry for C57BL/6 mice

|  | Test | **BUN** | **GLU** | **ALP** | **T-Pro** | **ALT** | **Cre** |
| --- | --- | --- | --- | --- | --- | --- | --- |
|  | Unit | mg/dl | mg/dl | IU/L | g/dl | IU/L | mg/dl |
| Untreated | Mouse 1 | 21 | 205 | 119 | 5.2 | 57 | 0.8 |
|  | Mouse 2 | 27 | 211 | 162 | 5.9 | 75 | 0.8 |
|  | Mouse 3 | 24 | 181 | 148 | 5.5 | 20 | 0.8 |
|  | Mouse 4 | 19 | 194 | 139 | 5.9 | 31 | 0.9 |
|  | Mouse 5 | 17 | 179 | 183 | 5.8 | 45 | 0.9 |
| 5% DMSO | Mouse 1 | 34 | 276 | 84 | 6.4 | 39 | 1 |
|  | Mouse 2 | 35 | 205 | 81 | 6.6 | 20 | 1 |
|  | Mouse 3 | 23 | 177 | 88 | 6.4 | 18 | 1 |
|  | Mouse 4 | 30 | 238 | 89 | 6.5 | 32 | 0.9 |
|  | Mouse 5 | 26 | 209 | 73 | 6.3 | 38 | 1 |
| SBPD-1 0.16 mg/kg | Mouse 1 | 28 | 188 | 94 | 6.1 | 21 | 1 |
|  | Mouse 2 | 37 | 248 | 108 | 6.2 | 31 | 1.2 |
|  | Mouse 3 | 37 | 285 | 94 | 5.9 | 22 | 1.1 |
|  | Mouse 4 | 27 | 219 | 96 | 5.9 | 14 | 1.1 |
|  | Mouse 5 | 28 | 189 | 85 | 6 | 28 | 0.8 |

Table S3. Complete Blood Count (CBC) for C57BL/6 mice

|  | Test | **WBC** | **RBC** | **HGB** | **HCT** | **MCV** | **MCH** | **MCHC** | **PLT** |
| --- | --- | --- | --- | --- | --- | --- | --- | --- | --- |
|  | Unit | 10^3^/mm^3^ | 10^6^/mm^3^ | g/dl | % | um^3^ | pg | g/dl | 10^3^/mm^3^ |
| Untreated | Mouse 1 | 5.4 | 7.98 | 13.8 | 36.6 | 92 | 34.8 | 75.8 | 1072 |
|  | Mouse 2 | 2.8 | 8.06 | 14.2 | 37 | 92 | 35.4 | 77 | 1062 |
|  | Mouse 3 | 3.6 | 7.66 | 13.4 | 35.4 | 92 | 35 | 75.8 | 1056 |
|  | Mouse 4 | 7.4 | 8.2 | 14.6 | 37.4 | 92 | 35.6 | 77.8 | 1084 |
|  | Mouse 5 | 3.4 | 7.7 | 13.8 | 36 | 94 | 35.8 | 76.4 | 826 |
| 5% DMSO | Mouse 1 | 8.2 | 7.64 | 12.8 | 33.4 | 88 | 33.4 | 76.2 | 1148 |
|  | Mouse 2 | 2 | 7.1 | 12 | 32 | 90 | 34 | 75.4 | 1004 |
|  | Mouse 3 | 3.2 | 6.68 | 12.2 | 31.8 | 96 | 36.6 | 77 | 866 |
|  | Mouse 4 | 2.4 | 8.12 | 13.4 | 36.2 | 90 | 33 | 74 | 1202 |
|  | Mouse 5 | 2.4 | 6.92 | 12 | 31.6 | 92 | 34.8 | 76.2 | 994 |
| SBPD-1 0.16 mg/kg | Mouse 1 | 1.6 | 8.08 | 13.4 | 36 | 90 | 33.8 | 75.8 | 1280 |
|  | Mouse 2 | 1.6 | 8.16 | 13.8 | 36.6 | 90 | 33.8 | 75.8 | 1068 |
|  | Mouse 3 | 1.8 | 7.02 | 12.4 | 32.2 | 92 | 35 | 76.4 | 1132 |
|  | Mouse 4 | 1.4 | 7.98 | 13.4 | 35.6 | 90 | 33.8 | 75.8 | 1280 |
|  | Mouse 5 | 2 | 8.02 | 14 | 35.8 | 90 | 34.8 | 78.2 | 1262 |

WBC: white blood cells, RBC: red blood cells, HGB: hemoglobin, HCT: hematocrit, MCV: mean corpuscular volume, MCH: mean corpuscular hemoglobin, MCHC: mean corpuscular hemoglobin concentration, PLT: platelet
